# Editorial Department

**Published:** 1861-11

**Authors:** George N. Burwell

**Affiliations:** Buffalo


BUFFALO, October 25, 1861.

DR. MINER—Dear Sir:—The following is the case of prolapse of the funis, related to you last evening, in which I followed Dr. T. Gaillard Thomas' mode of reduction. I comply with your request to furnish the case for publication, as an evidence of the great value attached to Dr. Thomas' recommendation, as a real improvement in the treatment of the accident, and for the purpose of calling attention to it in this practical manner.

Mrs. R. was taken in labor on the 22d inst., at 7 o'clock in the morning; there was not much pain until the water suddenly discharged at 8 o'clock, bringing down with it a large loop of the cord between the head and the pubis ; I was not sent for until this occurred ; it was full three quarters of an hour before I reached the house ; I found the patient in great trepidation, knowing that something was wrong, but not exactly its import; the pains were short and sharp, but not expulsive ; the os-uteri had nearly reached its full dilatation ; the tonic contraction of the womb in the interval of the pains was very marked, holding the head of the child firmly down into the superior strait, and from which any moderate lifting force with the hand was insufficient to disengage it sufficiently to permit the passage of a finger between the head and the bones of the pelvis; the pulsation of the cord was slow, (about 80,) thready and unequal when the pains were off, and totally interrupted when they were on.

I first attempted the replacement of the cord while my patient was lying on her back, but without success ; I had her then turn on to her knees, with the thighs at right angles to the body, and resting also on her elbows and shoulders on the pillows ; I introduced my hand and returned the

[illegible] ir which [illegible]

cord past the superior strait; but here, as when she was lying down, I encountered difficulty arising from the unusually firm contraction of the womb, keeping the head well engaged in the superior strait ; but after a little delay I noticed a relaxation of this contraction just before a pain, allowing the head to recede out of the strait, and taking advantage of this the cord was readily returned ; but a pain coming on forced it down as before ; again after some delay there was a partial relaxation and recession of the head, during which I passed the cord down beyond the promontory of the sacrum and well about the neck of the child ; it did not again come down ; the delay connected with these efforts, did not exceed fifteen minutes ; I kept the patient on her knees for a few pains after the return of the cord and then permitted her to lie down ; the head came slowly down and the labor was not completed for over an hour ; there was of course no further interference ; the child was still-born ; this I attribute entirely to the pressure, the cord was subjected to, for the hour after it came down, interfering with its circulation too greatly, and too long, for the safety of the child ; had I been present at the moment it came down, I could undoubtedly have replaced it much more quickly and readily than I did do, and have been successful in saving the child ; the success I had in returning the cord was entirely due to the position in which I placed the patient, and which I believe was first brought to the notice of the profession by Dr. Thomas. Due credit should be given him for his truly important recommendation.

Very truly yours, &c.,

GEORGE N. BURWELL.

